# The Discoloration of Copper Amalgam

**Published:** 1897-05

**Authors:** Kenneth W. Goadby


### Abstracts and Translations.

### THE DISCOLORATION OF COPPER AMALGAM.

BY KENNETH W. GOADBY.

Some time since, in anticipation of the paper just read, Mr. Badcock enlisted my help to determine if possible something definite as regards the discoloration copper amalgam undergoes in the mouth. Unfortunately the time at my disposal has been limited, so that the following results are rather fragmentary and incomplete. The points I have endeavored to solve are the chemical nature of the black and green discoloration, with the effect the latter conditions may have upon micro-organisms.

The literature of copper amalgam is most extensive and not a little contradictory, while no direct experiments seem to have been made with the well-known green-staining, although several observers have hazarded a guess. Other observers have made experiments as to its antiseptic properties, notably Miller, who placed pellets of copper amalgam upon plate-cultures, and found that the growth of colonies in their vicinity was inhibited, but at the same time unannealed soft gold had a similar effect. Now even if we allow that the copper does produce an antiseptic effect, it must be by the change of the metallic copper to some copper salt, but we cannot ascribe a like process to the gold cylinders. Under these circumstances it was thought desirable to confirm Miller's results. Plates of agar and gelatin were poured and small pellets of amalgam dropped on. In all the gelatin plates, five in number, a development of bacteria took place right up to the filling-material, which after a few days commenced to turn green. In three gelatin slant tubes a like effect was produced, the amalgam having no antiseptic action whatever. With the agar plates the series of events was even more interesting. Plates prepared in the above way after two or three days showed a distinct growth, the colonies sometimes stretching up to the mass of amalgam, and at other times some little distance away, making a well-marked zone; so far Miller is right. But the amalgam in all cases turned green and showed a ring of growth in its immediate vicinity; control tubes inoculated from this green zone developed rapidly upon agar, broth, or gelatin, while on agar and gelatin plates not inoculated with cultivations, no change whatever occurred in the amalgam, so I am able to point to conclusive evidence that the antiseptic effect of copper amalgam is an insignificant quantity. The explanation of the zone formed round the amalgam in which few or no colonies are to be seen is not difficult to explain when we consider the conditions. The medium agar is a damp one, and when a mass of material is placed upon its surface, the force of capillarity comes into play, a meniscus is formed, and the bacteria washed into it by the currents set up. Upon gelatin, which is a drier medium, this does not take place. The explanation appears to me to be perfectly in accordance with the facts, especially as colonies do eventually develop in the zone if inoculated when the agar has dried, while at times colonies may be seen below the surface also. Clearly, then, we cannot say that the amalgam exercises any antispetic effect upon plate cultures; but is its action upon fluid media of an antiseptic nature? The following experiments were made to determine this point:

A series of tubes of broth, saliva, and gelatin were inoculated with (1) cultures from the mouth direct; (2) streptococci (brevis); (3) impure cultures from the mouth. In all cases a development of bacteria took place. Thus, one to three pellets per ten cubic centimetres did not prevent the growth in any case, the gelatin tubes developing similarly in the broth and saliva under the same conditions; the controls grew perhaps a little more rapidly, but the difference was so small that we cannot attribute even very slight antiseptic effect to the amalgam under discussion.

Miller's experiments with copper sulphate are conclusive as far as they go, but as will be subsequently seen, experiment renders the presence of a sulphate improbable, consequently the antiseptic reactions of copper sulphate do not form an altogether satisfactory argument in dealing with amalgams containing copper.

The discoloration of Sullivan and allied fillings is too well known to require any explanatory remarks, while the division of the coloration into "black" and "green" varieties is a matter of clinical experience which requires no further comment, we may therefore at once discuss the production of the former. Several tubes of saliva were taken and small pellets of amalgam dropped in; a solution of sulphuretted hydrogen was next added, and the tubes placed in a chamber at the body temperature. Within a short space of time the familiar sooty appearance was to be seen, while in those tubes in which the solution was weakest a chocolate-brown color occurred exactly resembling a condition often noticed in the mouth. Upon gently rubbing the black deposit a shiny surface was exposed, due no doubt to the mercury set free. On again placing the mass in the solution, the surface became once more coated with the black sooty layer, placing beyond a doubt the question of sulphide. This is of course what was to be expected, the experiments simply giving direct evidence of a self-evident fact. In the control tubes the material remained quite bright, while in several cases in tubes inoculated with an extremely fetid culture of mouth bacilli the amalgam turned black, the culture having the extremely unpleasant smell of a dirty mouth. In tubes inoculated with pure cultures of the mouth streptococcus, this result was not forthcoming. De-

22

composition of food is a fruitful source of sulphuretted hydrogen, so that the series of events in the buccal cavity is evident, and although the percentage of sulphuretted hydrogen present at any given time would necessarily be small, the process would not thereby be suspended but only prolonged, the movements of the tongue and the rub of opposing teeth during mastication accelerating the process by exposing fresh surfaces to the denuding action; in fine, the condition may almost be compared to the gradual wearing away of rocks by the combined action of carbolic acid and rain.

I have made careful examination of the sooty deposit obtained in this way, and although copper sulphide is always present no reaction characteristic of mercury was to be obtained; it is not improbable that the mercury recombines with the more electronegative metal copper, eventually producing the curious pasty conditions sometimes met with. This hypothesis I cannot confirm by experiment, however, as in all cases a shiny condition was the only result. But the process just described is not the only method by which an amalgam may be disintegrated, the mouth organisms themselves produce chemical changes in the amalgam by which it is softened, eroded, and destroyed. To this we will recur later.

The second species of stain, often quite a dark green, was also investigated. The pigmentation has popularly been ascribed to copper sulphate, why is not quite clear, as many other copper salts are green, and at the same time the origin of a sulphate is, to say the least, obscure. On the other hand, more cautious observers have darkly hinted copper salts, thus leaving the field quite open.

Copper carbonate—that salt causing the green coloration of any article exposed for long to the air—would have been a far better theory, and it is most probable that this salt at times does occur, especially as the saliva normally contains sixty cubic centimetres of carbonic acid per one hundred cubic centimetres (Waller). However, I have attempted the determination of this salt by tests carried out in the following way. Teeth showing a well-marked condition of green stain were selected, ground up, and treated,—

(1) By prolonged boiling with sodium carbonate.

(2) Prolonged soaking in cold water.

(3) Dissolving in dilute acids.

(4) Dry reactions.

In the acid solutions the gelatin peptones formed so masked the other precipitates that this method was abandoned. Moreover, the small quantities of material at my disposal very much hampered the experiments.

The reactions of the filtrate from 2—1 were the same, and gave the following results:

(1) With barium nitrate a faint white precipitate, soluble in nitric acid.

(2) Silver nitrate gave a fairly well-marked precipitate, soluble in ammonia.

(3) Ammonium molybdate gave a slight coloration.

(4) Ferric chloride gave a blood-red coloration, soluble in hydrochloric acid.

(5) Heated with alcohol and sulphuric acid, acetic ether was given off.

Dry reactions.

(1) Original tooth fused with sodium carbonate on charcoal, and moistened with sulphuric acid on a bright silver surface, gave no black stain.

(2) Original tooth heated in tube with sodium carbonate and arsenic trioxide gave vapors of cacodyle.

There seems, therefore, no reason for supposing the salt to be copper sulphate, while the acetate reactions were well marked.

The next step was to produce the green coloration artificially.

Saliva tubes were taken and copper amalgam added, the tubes then being inoculated with cultures taken from the mouth, and a trace of lactose or dextrin added. In almost all cases, after forty-eight hours, a greenish tint made its appearance and gradually deepened till at the end of three weeks the coloration was most marked, the solution being acid.

These cultures gave the same reactions as the filtrates above mentioned, lactic acid being, curiously enough, not present so far as the ferric carbolate test is reliable. After a period of a month or six weeks little of the amalgam was left, a somewhat significant fact.

I have not at present sufficient material to determine the exact nature of the acetate formed under the above conditions, but from many considerations it is extremely probable that it is of an amidic nature, especially as glycocollate of copper is a well-known salt, crystallizing in blue needles and obtained by dissolving copper in a solution of glycocoll or amido-acetic acid; and once or twice I have seen needle-shaped crystals from the blue mass formed at the bottom of my cultures. I do not, however, wish to advance this as more than collateral evidence to a suggestion, as it is probable that other compounds are also formed,—*e.g.*, peptonate or albumate of copper, but I do state, most emphatically, that *a priori* remarks of the formation of copper sulphate are misleading and unscientific, and further, that its supposed antiseptic action depends, and must depend, upon the disintegration of a portion of the filling, a condition at once unsatisfactory and undesirable.

| | |
|---|---|
| (A) Saliva and Sullivan inoculated with culture from mouth (mixed). | (1) Good growth after twenty-four hours. Green coloration after seventy-two hours.<br>(2) Good growth after twenty-four hours. Green coloration after seventy-two hours.<br>(3) Good growth after twenty-four hours. Green coloration after seventy-two hours.<br>(4) Good growth after twenty-four hours. No color after three weeks.<br>(5) Good growth after twenty-four hours. Blackened after three weeks.<br>(6) Good growth after twenty-four hours. Blackened after three weeks. |
| (B) Broth and Sullivan inoculated with mouth culture (mixed). | (1) Good growth forty-eight hours. Amalgam blackened. Fetid.<br>(2) Good growth forty-eight hours. Amalgam blackened. Fetid.<br>(3) Good growth forty-eight hours. Amalgam blackened. Fetid.<br>(4) Good growth forty-eight hours. Slight coloration two weeks.<br>(5) Good growth forty-eight hours. Blackened.<br>(6) Good growth forty-eight hours. Blackened. |
| (C) Gelatin (hot incubator). From saliva mouth culture (impure). | (1) Good growth forty-eight hours. No coloration.<br>(2) Good growth forty-eight hours.<br>(3) Good growth forty-eight hours.<br>(4) Good growth forty-eight hours. Green coloration three weeks. |
| (D) Saliva and lactose and Sullivan inoculated from broth mouth culture (mixed). | (1) Good growth forty-eight hours. Green coloration at third day.<br>(2) Good growth forty-eight hours. Green coloration at third day.<br>(3) Good growth forty-eight hours. Green coloration at third day.<br>(4) Good growth forty-eight hours. Green coloration at third day.<br>(5) Good growth forty-eight hours. Green coloration at third day.<br>(6) Good growth forty-eight hours. Green coloration at third day. |

| | |
|---|---|
| (E) Saliva and dextrin and Sullivan inoculated from mouth culture saliva (mixed). | (1) Good growth forty-eight hours. No coloration.<br>(2) Good growth forty-eight hours. No coloration.<br>(3) Good growth forty-eight hours. Green coloration three days. |

A 1 and 2, B 4, C 4, D 1, 2, 3, 4, 5, 6, E 3 gave acetate reaction. N lactate.

| | |
|---|---|
| (F) Gelatin plate inoculated with mouth culture (impure). | (1) Green tinge to Sullivan colonies developed close up.<br>(2) Green tinge to Sullivan colonies developed close up. |
| Gelatin plate inoculated with streptococcus brevis (pure). | (1) Green tinge. No zone.<br>(2) Green tinge. No zone.<br>(3) Green tinge. No zone. |
| (G) Agar plate inoculated with mouth culture (impure). | (1) Green color well marked. Slight zone.<br>(2) Green color well marked. Zone.<br>(3) Green color well marked. Zone.<br>(4) Green color well marked. Colonies in zone under surface. |
| (H) Agar plate inoculated with pure culture streptococcus brevis. | (1) Green color well marked. Zone.<br>(2) Green color well marked. Zone.<br>(3) Green color well marked. Zone.<br>(4) Green color well marked. Zone. |

Broth tubes inoculated from green coloration of above plates developed a good growth in twenty-four hours.—*Transactions of the Odontological Society of Great Britain.*

